# Supplementary material for: Cell-to-cell infection by HIV contributes over half of virus infection
Source: eLife. 2015 Oct 6;4:e08150. doi: 10.7554/eLife.08150 (PMC4592948; doi:10.7554/eLife.08150)
Supplement: Supplementary file 1. — Technical details of MCMC computations. DOI: http://dx.doi.org/10.7554/eLife.08150.012 [file elife08150s001.docx]

**Supplementary file1: Technical details of MCMC computations**

Package FME [1] in Statistical software R [2] was employed to estimate posterior predictive parameter distributions. The delayed rejection and Metropolis method [3] as a default computation scheme equipped in FME was applied to perform MCMC computations. MCMC computations for parameter inference were implemented by using the pre-defined function modMCMC() in package FME, as introduced in Material and Methods. Convergence of Markov chains to a stationary distribution is required to ensure parameter sets are sampled from a posterior distribution. Only the last 15000 among 50000 chains were used as burn-in. The convergence of last 15000 chains was manually checked with figures produced by package coda [4] in statistical software R, a collection of diagnostic tools of MCMC computation. The 95% confidence interval drawn as a shadow region in each panel of **Fig. 2** is produced from 100 randomly chosen inferred parameter sets and corresponding model predictions.

**SI References**

1. K. Soetaert and T. Petzoldt. Inverse Modelling, Sensitivity and Monte Carlo Analysis in R Using Package FME, Journal of Statistical Software, 33(3) 1-28 (2010).
2. R Core Team (2014). R: A language and environment for statistical computing. R Foundation for Statistical Computing, Vienna, Austria.
3. H. Haario, M. Laine, A. Mira, and E. Saksman. DRAM: Efficient Adaptive MCMC, Statistics and Computing, 16, 339-354 (2006).
4. M. Plummer, N. Best, K. Cowles, and K. Vines. CODA: Convergence Diagnosis and Output Analysis for MCMC, R News, vol 6, 7-11 (2006).
