# Supplementary material for: Cell-to-cell infection by HIV contributes over half of virus infection
Source: eLife. 2015 Oct 6;4:e08150. doi: 10.7554/eLife.08150 (PMC4592948; doi:10.7554/eLife.08150)
Supplement: Supplementary file 2. — Estimated parameter values for Jurkat cell growth. DOI: http://dx.doi.org/10.7554/eLife.08150.013 [file elife08150s002.docx]

**Supplementary file 2: Estimated parameter values for jurkat cell growth**

Estimated parameter values of target cell growth dynamics in the static and shaking cell culture system

| Parameter Name | Symbol | Unit | Exp. 1 | Exp. 2 | Exp. 3 | Ave.$\pm$ S.D. |
| --- | --- | --- | --- | --- | --- | --- |
| Growth rate of jurkat cells in static cell culture | $g$ | day^-1^ | $0.59$ | $0.35$ | $0.47$ | $0.47\pm0.10$ |
| Growth rate of jurkat cells in shaking cell culture |  |  | $0.48$ | $0.67$ | $0.46$ | $0.54\pm0.09$ |
| Carrying capacity of flask in static cell culture | $T_{max}$ | ${10}^{6}\times$cells/ml | $1.50$ | $1.54$ | $1.49$ | $1.51\pm0.02$ |
| Carrying capacity of flask in shaking cell culture |  |  | $1.20$ | $1.21$ | $1.25$ | $1.22\pm0.02$ |
| Initial number of jurkat cells in static cell culture | $T(0)$ | ${10}^{5}\times$cells/ml | $5.62$ | $6.57$ | $5.93$ | $6.04\pm0.40$ |
| Initial number of jurkat cells in shaking cell culture |  |  | $6.10$ | $5.89$ | $5.30$ | $5.76\pm0.34$ |
