# Supplementary material for: Cell-to-cell infection by HIV contributes over half of virus infection
Source: eLife. 2015 Oct 6;4:e08150. doi: 10.7554/eLife.08150 (PMC4592948; doi:10.7554/eLife.08150)
Supplement: Supplementary file 3. — Estimated initial values for HIV-1 infection. DOI: http://dx.doi.org/10.7554/eLife.08150.014 [file elife08150s003.docx]

**Supplementary file 3: Estimated initial values for HIV-1 infection**

Estimated initial values for the static and shaking cell culture experiment

| Variable | Symbol | Unit | Exp. 1 | Exp. 2 | Exp. 3 | Ave.$\pm$ S.D. |
| --- | --- | --- | --- | --- | --- | --- |
| Initial number of target cells in static cell culture | $T(0)$ | ${10}^{5}\times$cells/ml | $3.75$^†^  ($1.44-8.93$)^‡^ | $4.10$  ($2.02-8.24$) | $4.04$  ($1.78-8.70$) | $3.96\pm1.83$^#^ |
| Initial number of target cells in shaking cell culture |  |  | $5.45$  ($5.45-5.45$) | $7.57$  ($1.53-27.54$) | $4.53$  (1.27$-15.34$) | $5.85\pm4.71$ |
| Initial number of target cells in static cell culture | $I(0)$ | ${10}^{3}\times$cells/ml | $4.43$  ($1.16-10.81$) | $3.12$  ($0.95-7.17$) | $3.68$  ($0.99-9.12$) | $3.74\pm2.21$ |
| Initial number of target cells in shaking cell culture |  |  | $0.01$  ($0.00-0.03$) | $0.02$  ($0.00-0.06$) | $0.03$  ($0.00-0.09$) | $0.02\pm0.02$ |
| Initial amount of HIV-1 in static cell culture | $V(0)$ | p24/ml | $295.62$  ($32.78-1215.93$) | $296.44$  ($33.41-1149.88$) | $319.97$  ($38.54-1321.26$) | $304.01\pm317.04$ |
| Initial amount of HIV-1 in shaking cell culture |  |  | $8.85$  ($1.10-22.47$) | $13.52$  ($0.88-50.02$) | $31.29$  ($2.33-128.56$ | $17.88\pm23.15$ |

^†^ Mean value

^‡^ 95% confidence interval

^#^ Average and standard deviation of merged values in experiment 1, 2, and 3
